# Water pollution and childhood leukaemia risk: a systematic review

**DOI:** 10.3332/ecancer.2026.2078

**Published:** 2026-02-20

**Authors:** Juan Carlos Nuñez-Enriquez, Daniela Medina-León, Diana Tinoco-Montejano, Karen Jacuinde-Trejo, Janet Flores-Lujano, Lissette Gómez-Rivera, Omar Chávez-Martínez, Francisco J García-Alvarado, Patricia Blanco-Padilla, Rosana Pelayo

**Affiliations:** 1 División de Investigación en Salud, Unidad Médica de Alta Especialidad Hospital de Pediatría ‘Dr. Silvestre Frenk Freund’, Centro Médico Nacional Siglo XXI, Instituto Mexicano del Seguro Social, Mexico City 06720, Mexico; 2 Facultad Mexicana de Medicina, Universidad La Salle, Mexico City 14000, Mexico; 3 Departamento de Medicina, Facultad de Estudios Superiores Zaragoza, Universidad Nacional Autónoma de México, Mexico City 09230, Mexico; 4 Escuela Nacional de Medicina y Homeopatía, Instituto Politécnico Nacional, Mexico City 07320, Mexico; 5 Unidad de Investigación Médica en Epidemiología Clínica, Unidad Médica de Alta Especialidad Hospital de Pediatría ‘Dr. Silvestre Frenk Freund’, Centro Médico Nacional Siglo XXI, Instituto Mexicano del Seguro Social, Mexico City 06720, Mexico; 6 Centro de Documentación en Salud, Dirección de Educación e Investigación en Salud, Unidad Médica de Alta Especialidad Hospital de Oncología, Centro Médico Nacional Siglo XXI, Instituto Mexicano del Seguro Social, Mexico City 06720, Mexico; 7 Coordinación de Investigación en Salud, Unidad de Educación e Investigación, Instituto Mexicano del Seguro Social, Mexico City 06720, Mexico; 8 Departamento de Investigación en Salud, Unidad Médica de Alta Especialidad No. 71, Instituto Mexicano del Seguro Social, Torreón 27000, Mexico; 9 Jefatura de Laboratorios de la División Ciencias de la Salud. Instituto de Estudios Superiores de Tamaulipas, IEST - ANÁHUAC, Tamaulipas 89605, Mexico; 10 Laboratorio de Citómica del Cáncer Infantil, Centro de Investigación Biomédica de Oriente, Instituto Mexicano del Seguro Social, Puebla 74360, Mexico; 11 Unidad de Educación e Investigación, Instituto Mexicano del Seguro Social, Mexico City 06720, Mexico; a https://orcid.org/0000-0002-8070-9727; b https://orcid.org/0009-0005-2922-6219; c https://orcid.org/0009-0003-8688-2330; d https://orcid.org/0009-0005-2584-9443; e https://orcid.org/0000-0003-0727-2837; f https://orcid.org/0000-0002-3337-1575; g https://orcid.org/0000-0003-2633-1898; h https://orcid.org/0000-0002-3760-1876; i https://orcid.org/0009-0004-4836-6041; j https://orcid.org/0000-0002-8070-9727

**Keywords:** water pollution, toxic metals, pesticides, childhood leukaemia, systematic review

## Abstract

Water pollution represents a major global health concern, especially in low- and middle-income countries where toxic metals (TMs) and pesticides can contaminate drinking water through industrial, agricultural and urban activities. Children are particularly susceptible due to their developing physiology and higher water intake relative to body weight. This review aims to explore the association between exposure to TMs and pesticides in drinking water and the risk of childhood leukaemia (CL), highlighting the broader significance for environmental health and child safety. A structured search in PubMed, Scopus and Google Scholar (2001–2024) identified studies on acute lymphoblastic leukaemia or acute myeloid leukaemia in individuals under 20 years of age, assessing exposure to trace metals or pesticides via drinking water. Observational designs were included, excluding studies unrelated to water exposure or lacking paediatric data. Records were screened and reviewed independently by four authors. Findings were heterogeneous, with several studies suggesting potential links between specific contaminants like arsenic, hexavalent chromium, pentachlorophenol and certain pesticides and an increased risk of leukaemia in children, while others found no significant associations and noted methodological challenges such as small sample sizes and difficulties in exposure measurement. Although current evidence remains inconclusive regarding a direct causal relationship, this review underscores the need for rigorous, long-term research to clarify the role of waterborne pollutants in CL and to guide public health strategies.

## Background

Water pollution (WP) is a significant global public health issue linked to cancer development [1–5]. Toxic metals (TM) and pesticides, detected in water from industrial, agricultural and domestic sources [1, 2, 6, 7], pose risks to children due to their developing physiology and longer life expectancy [8–12]. Childhood leukaemia (CL), mainly acute lymphoblastic leukaemia (ALL) and less commonly acute myeloid leukaemia (AML), is one of the most frequent paediatric cancers [13]. Although some studies associate exposure to TM and pesticides in water—like arsenic (As), lead (Pb), cadmium (Cd) and certain pesticides—with increased leukaemia risk, evidence remains inconsistent [14–19]. In Latin America, WP is severe, with elevated levels of TM and banned pesticides, stemming from natural sources or human activities, but monitoring is often limited, creating significant data gaps [20–25]. Assessing whether waterborne contaminants contribute to CL is crucial, especially in low- and middle-income countries with weaker environmental controls and higher CL incidence [20–22, 26, 27]. This systematic review examines literature from 2001 to 2024 to evaluate links between TM and pesticide exposure in water and CL risk, aiming to synthesize evidence, identify gaps and inform future research and public health actions.

## Methods

A structured search strategy and selection criteria were applied to ensure the inclusion of relevant and methodologically sound studies.

Studies were included if they reported cases of ALL or AML in populations under 20 years of age, regardless of whether adult populations were also included in the study. That is, studies involving mixed-age populations were eligible, provided that data on individuals under 20 years were clearly analysed.

Eligible studies assessed exposure to trace metals (e.g., As, Cd and Pb) or pesticides via drinking water or directly related environmental pathways. We considered observational designs such as case-control, systematic reviews, ecological or geospatial analyses.

Exclusion criteria included studies focusing exclusively on chronic leukaemias or on populations over 20 years of age without clear inclusion of paediatric or adolescent subgroups. Studies were also excluded if the exposure pathway was unclear, unrelated to water or if they lacked sufficient information on the contaminants or exposure mechanisms.

A targeted literature search was conducted in PubMed, Scopus and Google Scholar. The most recent search was performed on May 15, 2025. The search strategy was structured around three core concepts: leukaemia, water contamination and paediatric population. Controlled vocabulary (MeSH, DeCS) and free-text terms were combined using Boolean operators and adapted to the syntax of each database.

### Key search terms included

- Leukaemia: ‘Leukaemia’[Mesh], ‘Leukaemia, Lymphoid’[Mesh], ‘Leukaemia, Myeloid’[Mesh], leukaemia
- Water contamination: ‘Water Pollutants’[Mesh], ‘Drinking Water’[Mesh], water contamination, toxic water
- Population: ‘Child’[Mesh], ‘Adolescent’[Mesh], ‘Infant’[Mesh], child, adolescent, teen, paediatric

Custom searches were also conducted for specific contaminants (e.g., As, benzene and nitrates) and gray literature was retrieved through flexible queries in Google Scholar (e.g., ‘water contamination’ AND leukaemia AND children), applying manual filters for language (English/Spanish) and publication years (2001–2024).

### Study selection and data summary

**Figure 1.**
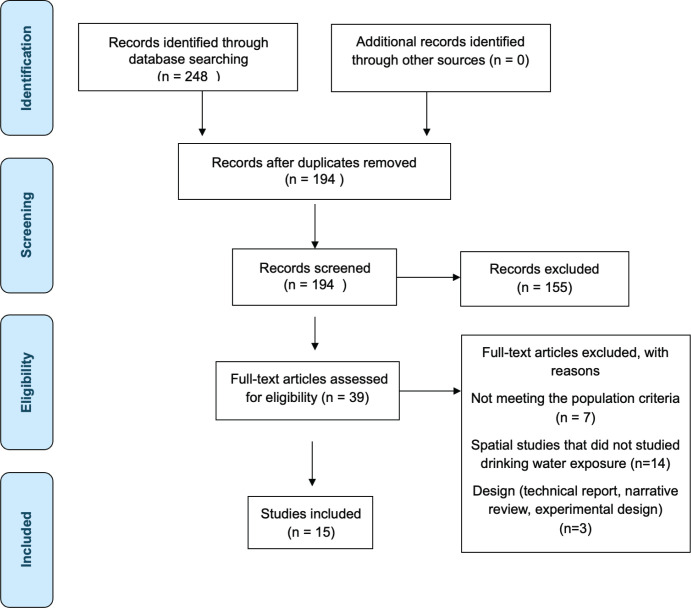
PRISMA flow diagram.

All records were independently reviewed by four authors (JFL, DML, DTM, KJT) in two phases: title/abstract screening followed by full-text review. Studies were selected based on relevance and methodological clarity. Disagreements were resolved by consensus (Figure 1).

## Results

A total of 15 studies were included in this systematic review, covering a range of geographical regions including North America, Europe, Asia and Africa. These studies were conducted between 1980 and 2023 and employed various study designs, including case-control, ecological and archival research, to investigate the potential association between WP by TM and pesticides and the risk of CL. The sample sizes ranged from small case-control studies (less than 100 participants) to larger ecological studies involving tens of thousands of individuals.

The studied populations varied, with most studies focusing on children or individuals under 20 years of age, though a few also included adults. The primary outcome assessed across these studies was CL, with some studies also examining other hematological malignancies such as lymphoma and myeloma. The cancers investigated were predominantly childhood ALL and other forms of leukaemia, though some studies also considered broader cancer types such as non-Hodgkin lymphoma, multiple myeloma and chronic lymphocytic leukaemia.

The studies primarily focused on exposure to specific substances in DW, such as As, chromium (hexavalent), radon, volatile organic compounds and various pesticides, including herbicides like atrazine, simazine and alachlor. Exposure mechanisms were primarily linked to DW, with a few studies also considering oral ingestion through food, geophagia and residential proximity to contaminated areas [28–32]. The exposure assessment methods varied, with some studies measuring contaminant concentrations directly in water samples using techniques such as liquid scintillation, atomic absorption spectrometry or gas chromatography, while others used geographic models or databases to estimate exposure levels [33, 34].

The primary aim of most studies was to evaluate whether there was an increased risk of CL or other cancers in populations exposed to these contaminants [28, 31, 32, 35–37]. Some studies sought to identify specific environmental factors or contaminants responsible for observed cancer clusters in certain regions, while others aimed to investigate the general health risks associated with long-term exposure to polluted water sources [29, 30, 33, 34, 38, 39].

In terms of study databases, several studies utilised national or regional environmental health databases, including public water system data, government environmental records and cancer registries [29, 30, 32, 40]. Some studies also used data from more specific sources, such as municipal water records or specialised cancer registries, to assess exposure and cancer incidence [28, 31, 39]. The variety in methodologies and data sources across the studies reflects the complexity of accurately assessing the link between WP and CL risk. Despite the heterogeneity in study designs, a consistent pattern emerged suggesting a potential association between exposure to TM and pesticides in DW and increased leukaemia risk in children.

To provide a concise overview of the studies included in this review, Table 1 summarises the main characteristics of the 15 analysed articles. It describes the study design, population, type of leukaemia, exposure source, key findings and a brief quality commentary, allowing a comparative assessment of the methodological strengths and limitations of each investigation (Table 1).

## Abbreviations

AL – Acute Leukaemias, ALL – Acute Lymphoblastic Leukaemia, AML – Acute Myeloid Leukaemia, As – Arsenic, ASR – Age-Standardised Rate, BbF – Benzo[b]fluoranthene, BkF – Benzo[k]fluoranthene, Be – beryllium (BrDCM) Bromodichloromethane, CHBr₃ – Bromoform (C₄H₆) 1,3-Butadiene, C₆H₆ – Benzene, C₈H₈ – Styren, CBPR – Community-Based Participatory Research, Cd – Cadmium, CHR – Chrysene, CC´s – childhood cancer(s), CI – Confidence Interval, CL – Childhood Leukaemia, CHCl₃ – chloroform, CO – Carbon Monoxide, Cr – Chromium, CrVI) – Hexavalent Chromium, DCE – trans-1,2-Dichloroethylene, DBA – Dibenz[a,h]anthracene, DE – Diesel Exhaust, DW – Drinking Water, EtOH – Ethanol, EtO – Ethylene Oxide, FI-HG-AAS – Flow Injection-Hydride Generation-Atomic Absorption Spectrometry, GC-MS/MS – Gas Chromatography–Mass Spectrometry, GIS – Geographic Information Systems, HCHO – Formaldehyde, Hg – Mercury, HL – Hodgkin Lymphoma, HM – Haematological Malignancy, HPC – Heptachlor, IARC – International Agency for Research on Cancer, ICP-MS – Inductively Coupled Plasma-Mass Spectrometry, IP – Indeno[1,2,3-cd]pyrene, MDS/MPS – Myelodysplastic/Myeloproliferative Syndrome, MM – Multiple Myeloma, N₂H₄ – Hydrazine, NHL – Non-Hodgkin Lymphoma, NH₄⁺ – Ammonia, NO₂ – Nitrogen Dioxide, NO₃ – Nitrates, NTDs – Neural Tube Defects, NTP – National Toxicology Program, OR – Odds Ratio, P – P Value, PAHs – Polycyclic Aromatic Hydrocarbons, PCP – Pentachlorophenol, Pb – Lead, PCE – Tetrachloroethylene, PM₁₀ – Particulate Matter 10, PO – 1,2-Propylene Oxide, Rn – Radon, RRs – Relative Risks, Se – selenium, SDs – standard deviations, SEER – Surveillance Epidemiology and End Results Program, SiO₂ – Quartz, SIRS – Standardised incidence ratios, SMRs – Standardised Mortality Ratios, SO₂ – Sulfur Dioxide, SRRs – Standardised Rate Ratios, TCE – Trichloroethylene, THMs – Trihalomethanes, TNT – Trinitrotoluene, U – Uranium, UO&G – Unconventional Oil and Gas, USA – United States of America, VOCs – Volatile Organic Compounds, y – years, Zn – Zinc

## Discussion

The environmental factors influencing CL have been widely debated, with varying findings across different regions and exposure types. This systematic review examined the relationship between environmental contaminants, particularly in DW and CL. The findings from the included studies are diverse, with both significant associations and inconclusive results, pointing to the complexity of linking environmental exposure to leukaemia risk.

### Water contaminants and CL risk

Several studies have highlighted the role of DW contaminants in the development of CL. Infante-Rivard *et al* [35] found an increased risk of ALL associated with exposure to trihalomethanes and metals like Cd and As in DW in Quebec, Canada. However, they acknowledged the challenges posed by measurement variability and exposure misclassification. Similarly, Moore *et al* [28] in Nevada found no significant association between As levels in DW and CL, suggesting that the relationship might not be as straightforward or might be influenced by other factors [28]. Costas *et al* [39] investigated potential associations in Woburn, USA, and found a non-significant link between contaminated water exposure during pregnancy and leukaemia diagnosis, although a significant dose-response relationship was observed for maternal exposure during pregnancy.

These findings underscore the complexities of exposure assessment in water contamination studies. The potential for misclassification and the difficulty in accurately measuring long-term exposure levels pose substantial challenges. Further, the studies often relied on ecological or case-control designs, which have limitations such as recall bias and limited power to detect weak associations.

### Chemical exposure and childhood cancer

In addition to waterborne contaminants, industrial chemicals have been a focus of research. García-Pérez *et al* [29] conducted an ecological study in Spain and found an increased mortality risk from leukaemia in populations near metal industries that released pollutants into the air. This result aligns with earlier studies by Thorpe and Shirmohammadi [32], who reported potential associations between childhood cancers and the presence of pesticides, particularly herbicides like atrazine and simazine, in groundwater. The study highlighted that childhood cancers, particularly leukaemia, may be influenced by a combination of chemical exposures, but also noted that genetic factors and other environmental exposures, such as household pesticide use, could confound the results [32].

### Geographic and temporal variability

**Figure 2.**
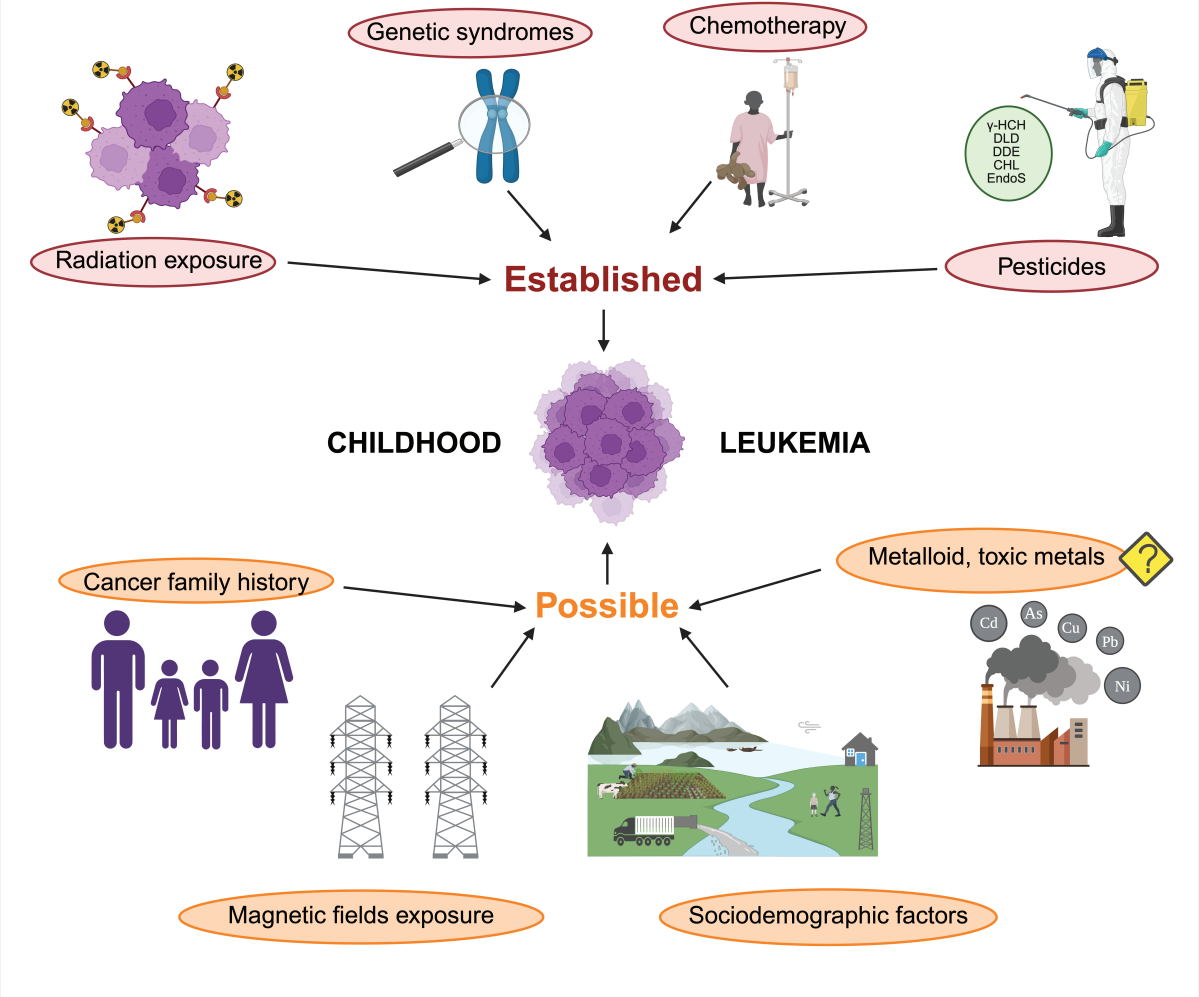
Established and possible risk factors for the development of leukaemia [47–49].

Several studies have examined the spatial and temporal dimensions of environmental exposure to understand how localised pollution may contribute to CL. Thompson *et al* [31] found that certain watersheds in Texas were associated with higher risks of specific childhood cancers, including leukaemia, while García-Pérez *et al* [29] noted a dose-response relationship with proximity to industrial installations. This geographic variability suggests that localised environmental factors, such as the release of toxic substances by industries, may be a key contributor to increased cancer risks (Figure 2).

Furthermore, studies like Ruckart *et al* [41] and Elliott *et al* [38] have examined the role of specific chemical contaminants, such as tetrachloroethylene and benzene, in CL. While the evidence is still inconclusive, they emphasize the need for more comprehensive, longitudinal studies that include better exposure assessment methods and consider additional factors such as genetic predisposition and lifestyle.

### Molecular interactions

**Figure 3.**
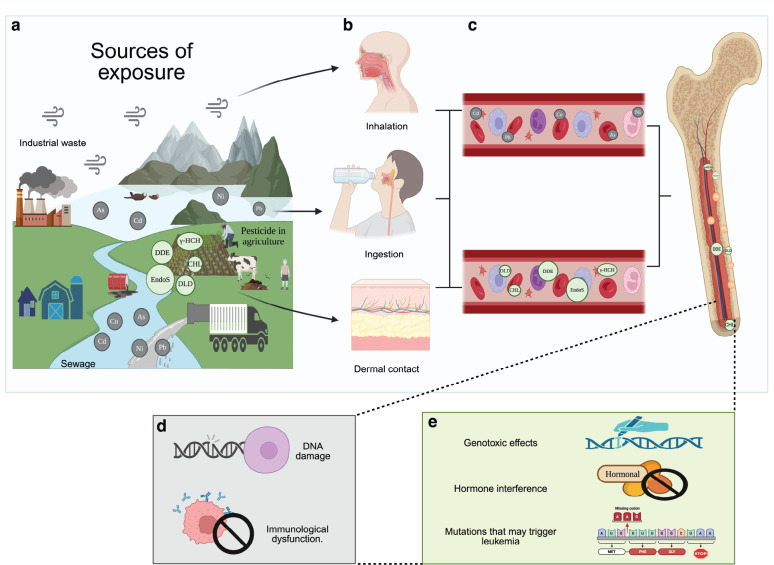
Main contact pathways of contaminated water by TM and pesticides related with CL development. (a) WP with TM and pesticides by sewage, industrial waste and pesticide use in agriculture. (b) The contaminants may enter into the human body through different ways: inhalation, ingestion and/or dermal contact. (c) Circulation of the toxic agent in the vascular system up to the bone marrow and their absorption. (d) Cellular impact of TM in hematopoietic cells potentially involved to the development of CL. (e) Described possible effects of pesticides exposure in the hematopoietic cells related to the development of CL [50, 51].

In recent years, exposure to water contaminated with different TM and pesticides, especially organophosphates and organochlorines, has been increasing [42]. However, the consequences derived from their consumption and their possible relationship with the development of CL

have not been explored in depth. Among what has been described, there have been a few environmental factors reported to be linked with the development of different types of cancer. Some examples of these established risk factors are radiation exposure, genetic syndromes, chemotherapy and pesticides (e.g., benzene) (Figure 3). On the other hand, it has been suspected that TM and other types of pesticides interact directly with hematopoietic stem cells, altering their capacity to proliferate and differentiate [43].

**Figure 4.**
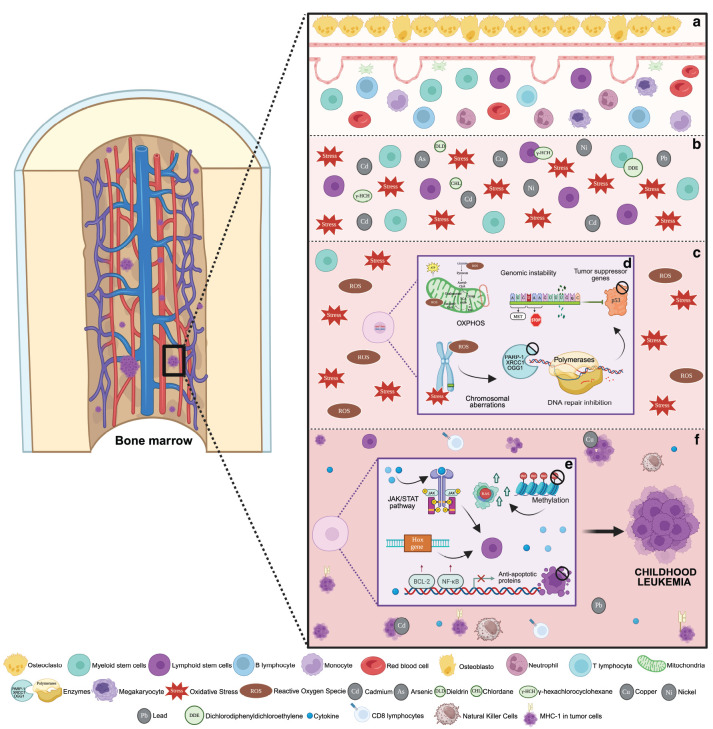
Molecular pathways disrupted by TM and pesticide exposure potentially linked to leukaemia. (a) Normal hematopoiesis. (b) Bone marrow contamination with TM and pesticides generating cellular stress. (c) Alteration of the microenvironment by oxidative stress with the generation of free radicals and reactive oxygen species. (d) DNA damage generating mutations in critical genes (TP53), chromosomal aberrations such as translocations and inhibition of enzymes involved in DNA repair (PARP-1, OGG1, XRCC1 and polymerases). (e) Modification of hematopoietic precursors by alteration of signaling pathways such as JAK/SAT and alteration in HOX gene expression. Interruption of cell apopotosis due to activation of BCL-2 and NF-KB. Global DNA hypomethylation activating oncogenes such as RAS. (f) Bone marrow with leukaemic clones secondary to immunosuppression and evasion of the immune response due to reduced expression of MHC-I in tumour cells [37–39].

Also, both pesticides and TM have been related to the alteration of the hematopoietic microenvironment (Figure 4) [44–46]. This alteration is generated by the hyperproduction of reactive oxygen species, producing oxidative stress. This causes damage to cellular DNA, including double-strand breaks and mutations in genes critical for hematopoiesis [44–46].

This microenvironment favours the formation of free radicals, which modify or hinder the function of various enzymes and isomerases involved in DNA replication or DNA repair enzymes, such as PARP-1 and OGG1. In consequence, the accumulation of mutations triggers chromosomal aberrations, among the most common, translocations, resulting in genomic instability [45, 47, 48].

Similarly, epigenetic alterations occur, affecting the cell cycle. First, by modulating DNA methylation and histone acetylation, resulting in a decrease in the expression of tumour suppressor genes and activating oncogenes. Second, by altering cell signaling pathways such as or NF-κB, which directly alter the proliferation and differentiation of hematopoietic precursors or by altering genes such as BRCA1 and TP53, key in tumour suppression [49, 50].

All these mechanisms chronically generate an alarming immunosuppression, since the levels of natural killer and CD8+ T lymphocytes are reduced, thus allowing the proliferation of leukaemic cells [51] (Figure 4).

### Challenges and limitations in environmental exposure research

Despite the vast body of literature on environmental contaminants and CL, many studies, including those by Oller-Arlandis and Sanz-Valero [37] and Cheng *et al* [33], face significant methodological limitations. These include the reliance on ecological data, small sample sizes and lack of direct exposure measurements. Most studies rely on residential proximity to contamination sources or indirect exposure estimates, which can lead to exposure misclassification. Furthermore, the limited availability of data on individual-level factors like diet, occupation and personal habits makes it difficult to establish clear causal links.

A notable example of these challenges is the study by Ruckart *et al* [41], which found associations between contaminated DW and childhood cancers but acknowledged the difficulty in controlling for all potential confounding variables, including the lack of detailed information on household water usage and occupational exposures.

### Future directions

Future research should focus on more robust study designs, including cohort studies that can better assess individual exposure and account for confounding variables. The use of biomarkers and more precise exposure assessments, including the measurement of specific contaminants over time, could significantly enhance the accuracy of findings. Additionally, the role of genetic factors in modulating the effects of environmental exposures should be explored more thoroughly, as genetic susceptibility may play a crucial role in determining the risks of leukaemia from environmental pollutants.

Longer-term, multi-center studies with diverse populations, particularly in low- and middle-income countries, are needed to strengthen the evidence and address the geographic and temporal variability in exposure patterns. Moreover, integrating environmental monitoring data with clinical cancer registries would provide a more comprehensive understanding of the relationship between environmental exposures and CL.

In conclusion, while evidence of a link between environmental contaminants in DW and CL is accumulating, further studies are needed to clarify the strength of these associations and to overcome the methodological challenges of exposure assessment and confounding factors. The complexity of these relationships requires ongoing investigation and a multidisciplinary approach to understand how environmental factors contribute to childhood cancer risk.

## List of abbreviations

ALL – acute lymphoblastic leukaemia, AML – acute myeloid leukaemia, As – arsenic, Cd – cadmium, CL – childhood leukaemia, DeCS – Descriptores en Ciencias de la Salud, IMSS – Instituto Mexicano del Seguro Social, MeSH – Medical Subject Headings, Pb – lead, PRISMA – Preferred Reporting Items for Systematic Reviews and Meta-Analyses, TM – toxic metals, UMAE – Unidad Médica de Alta Especialidad, USA – United States of America, WP – water pollution.

## Conflicts of interest

The author(s) declare that they have no conflicts of interest.

## Funding

The author(s) declare that financial support was received for the research, authorship and/or publication of this article. This work was supported by grants from the Secretaría de Ciencia, Humanidades, Tecnología e Innovación (SECIHTI), previously known as the Consejo Nacional de Humanidades, Ciencias y Tecnologías (CONAHCYT) FORDECYT-PRONACES 302994 to RP and FORDECYT-PRONACES 303019 to JN-E.

## Figures and Tables

**Table 1.** Epidemiological evidence of childhood leukaemia and exposure to DW contaminants: summary table.

Author (Year)	Region/Country	Study design/Period	Sample/Population	Cancer type	Substances identified	Objective	Measurement	Results	Limitations
Infante-Rivard *et al* [19]	Québec, Canada	Case-control/1980–1993	491 cases/controls (0–19 years)	ALL	THMs (CHCl_3_, CHBr_3_, BrDCM, Chlorodibromomethan), As, Cd, Cr, Pb, Zn, NO_3_.	Assess DW contaminants and CL	Multi-source matrix included 327 rows, one for each distinct municipality for which data from any source were available and 11 columns for the following conaminants: four specific THMs, total THMs, five metals, and NO_3_ from 1970 to 1993. To fill the cells from the matrix, measurements were made from all three sources (municipal records, Ministry of Environment data, and tapwater survey) when available.	Slightly increased risk of childhood ALL with postnatal exposure to THMs, As, and Cd; no strong association during prenatal exposure.	Misclassification, no personal water usage data, spatial exposure uncertainties, and the ecological nature of some comparisons
Moore *et al* [28]	Nevada, USA	Ecological/1979–1999	17 counties (estimated population of 327,947 children and young adults between 0 and 19 years)	AL	As	Test As in DW might cause increases in leukemia	County-level As	No association found between As levels in DW and CL incidence in Nevada.	Small sample, limited exposure data
Costas *et al* [39]	Woburn, USA	Case-control/1969–1989	21 cases, 37 controls (0–19 years)	AL	Industrial pollutants: Greenhouses, leather manufacturers, As, TNT, based pesticides, textiles, paper, and animal glues.	Identify contamination link to cluster of leukemia cases	Residence-based exposure scoring	Non-significant associations were found for prenatal exposure; however, a significant dose–response relationship during pregnancy was observed, suggesting that maternal exposure during pregnancy may increase the risk of CL, although with considerable uncertainty.	Small sample, recall bias
Seiler [52]	Fallon, USA	Longitudinal observational/ 1989–2001	100 wells, residents of Fallon, Nevada, 16 cases (0–19 years)	ALL	Rn-222, U, metals, Gross-alpha and gross-beta radioactivity, pesticides, VOCs, Cd, Cr, Pb, Se, Be, As, Rn	Evaluate current and past DW quality in Fallon and compare well water used by leukemia case families to that of another resident.	Rn-222: liquid scintillation, U isotopes: pulsed-laser phosphorimetry following ASTM method D3972-97, Gross-alpha and gross-beta radioactivity: EPA method 900.0, trace elements (As, Be, Cd, Cr, Pb and Se): ICP-MS FI-HG-AAS and ICP-MS	No significant differences in contaminant levels were observed between affected and control wells, indicating that DW quality was not associated with the reported leukemia cluster.	No causal evidence, analytical and sampling method variations, well selection bias, spacial and depth variability, limited scope of contaminants analyzed
Thorpe and Shirmohammadi [32]	Maryland, USA	Spatial analysis/1992–1998	A total of 689 cases, including 293 leukemia cases (0–17 years)	CCs	Herbicides, atrazine, simazine, alachlor, and metolachlor and NO_3_	Spatial distribution versus CC´s, and correlations between them and the distribution of NO_3_ and herbicides	ArcView buffers	Possible associations between low-level herbicide and nitrate exposure and CC, indicating potential synergistic environmental effects.	Many confounders, proximity bias
García-Pérez *et al* [29]	Spain	Ecological/1994–2003	8,073 towns were analysed, with leukaemia-related mortality in Spain amounting to 40,050 deaths between 1994 and 2003	AL	NH_4_^+^, As, C_6_H_6_, Cd, chlorides,Cr, Cu, cyanides including hydrogen cyanide, DCM, fluorides, halogenated organic compounds, Pb, Hg, methylene chloride, Ni, NO₂, PAHs, total organic carbon, Zn, PCE, TCE, VOCs, PM₁₀, SO₂, CO	Assess leukemia mortality near metal industries	Distance to source	Modest but statistically significant increases in leukemia-related mortality were observed among populations residing near metal production and processing installations, particularly those emitting airborne pollutants such as dioxins, chlorinated solvents, and metalworking fluids, indicating a higher risk with closer residential proximity.	Used mortality instead of incidence; may underestimate risk due to high survival in children; ecological fallacy; exposure misclassification; limited to 2001 emission data
Thompson *et al* [31]	Texas, USA	Spatial analysis/1990-2005	3,718 cases, including 1,248 leukemias (0–13 years)	CC´s	Pesticides	Estimate the risk of cancer to a child when the mother’s living location at the time of birth was located within speciﬁc watersheds	GIS watershed location	Nine watersheds in Texas showed a high probability of increased risk for specific types of childhood cancer, including renal cancer, acute lymphoid leukemia, and atypical leukemias, suggesting a possible link between DW contaminants and cancer risk.	Misclassification, no direct measures
Linos *et al* [30]	Greece	Ecological/1999–2009	A total of 118 cancer deaths, of which seven were due to leukemia (0–80 years)	All types of cancer	Cr(VI)	Study mortality from Cr(VI) DW use	-------	Increased cancer mortality was observed in the exposed area, supporting the classification of DW contaminated with (Cr VI) as a potential human carcinogen.	Short follow-up, no biomarkers, and the ages of individuals who died from leukemia were not specified.
Paul *et al* [36]	India	Case-control	70 cases and 30 controls, of which 49 were leukemias (10–60 years)	Leukemia variants (ALL, AML, CML, CLL. HL, NHL, MM, MDS)	As	Assess blood cancer–As link	Biological tissue testing: Short-term leucocyte cultures: Method of Moorhead PS, modified by the method of Sharma and Talukder Complete haemogram: Sysmex K-2000, automated cell counter Sysmex K2000, automated cell counter Estimation of As concentration: FI-HG-AA at 327 nm was used for estimation of As in the collected biosamples. A Perkin-Elmer model 3100 AAS equipped with a Hewlett-Packard-Vectra 386/25N computer with GEM software, Perkin-Elmer EDL System-2, As lamp (lamp current 400mA) was used for this purpose.	Higher As concentrations in biological tissues correlated with chromosomal aberrations and leukemia, supporting a possible causal link.	SES confounding, no molecular details, and the age of children with leukemia was not specified.
Oller-Arlandis and Sanz-Valero [37]	Spain	Systematic review	20 studies including 2,686 children and young adults diagnosed with CL (0–19 years)	Cancer	C_6_H, NO_3_, CHCl_3_, THMs, BrDCM, agricultural pesticides, perchlorate, Cr, trichloroethane,	To evaluate the association between exposure to major chemical contaminants in DW and increased cancer cases in children <19 years of age.	-------	Evidence from 20 studies was heterogeneous; some showed dose–response patterns, but overall findings remain inconclusive.	Studies heterogeneity
Ruckart *et al* [41]	North Carolina, USA	Case-control/1968–1985	51 cases and 526 controls, of which 11 were leukemias (<20 years)	Hematologic cancers	PCE, TCE, C_6_H_6_, Vinyl Chloride, DCE	Link DW toxins to child cancer	Water model estimates	Exposure to benzene and TCE during pregnancy showed increased odds for leukemia and neural tube defects, although based on few cases.	Small N, model limitations
Elliott *et al* [38]	USA	Systematic review/2015	49 studies (1,177 water contaminants and 143 air pollutants)	AL/lymphoma	C_6_H_6_, C_4_H_6_,Cd, EtOH, EtO, HCHO, SiO₂, DBA, DCM, PCE, PO, BbF, BkF, HPC, N₂H_4_, IP,C₈H₈, DE, PAHs, CHR	Assess UO&G carcinogenicity	-------	Review identified several UO&G-related chemicals classified as carcinogenic or leukemogenic, but direct human exposure data remain limited.	No exposure data, ecological designs
Cheng *et al* [33]	China	Ecological study/2009–2012	6,750 cases (1–98 years)	All types of cancer	PCP	Explore the cancer risks of long-term community-level PCP exposure	GC-MS/MS analysis	Cancer incidence was markedly elevated in the high-exposure group, particularly for leukemia (SRR = 5.93, 95% CI: 5.24–6.71) and malignant lymphoma (SRR = 2.27, 95% CI: 2.10–2.54), suggesting that prolonged PCP exposure may increase cancer risk.	Small sample size, unclear mechanisms, inadequate control of bias, variability in individual susceptibility, and the exact number of leukemia cases was not specified.
Danjou *et al* [40]	South Africa	Case-series/2014–2015	556 cancer patients, of which 62 were leukemias (18–90 years)	AL/lymphoma, myeloma	U	Describe cases and exposure routes	----.	NHL (37.6%) was the most frequent cancer, followed by leukemia (32.8%), HL (13.8%), and myeloma (13.2%). Leukemia predominated among individuals aged 18–30 and over 60, while myeloma increased with age. A small proportion of patients (6.3%), all men, had worked in mines and were mainly diagnosed with leukemia, myeloma, or NHL. Although piped water was the primary source during childhood (76.6%) and adulthood (97.9%), some individuals also consumed borehole, well, or river water, particularly in childhood, indicating potential contamination routes. Government reports corroborate that rivers and streams in gold mining areas—used for drinking and cooking—are contaminated with U.	No geocoding, referral inconsistencies, and the age of leukemia cases was not specified.
Ashwood et al [53]	Alabama, USA	Survey/2017–2021	515 households	Cancer	Metals, VOCs, Rn	CBPR in cancer cluster research	Water sampling, survey	DW contamination was linked to leukemia cases within the community, and subsequent local action and engagement contributed to addressing the identified environmental health concerns.	No historical comparison, geocoding issues
